# Single Aggressive Interactions Increase Urinary Glucocorticoid Levels in Wild Male Chimpanzees

**DOI:** 10.1371/journal.pone.0118695

**Published:** 2015-02-25

**Authors:** Roman M. Wittig, Catherine Crockford, Anja Weltring, Tobias Deschner, Klaus Zuberbühler

**Affiliations:** 1 Max Planck Institute for Evolutionary Anthropology, Department of Primatology, Leipzig, Germany; 2 University of St. Andrews, School of Psychology and Neurobiology, St. Andrews, United Kingdom; 3 Budongo Conservation Field Station, Masindi, Uganda; 4 University of Neuchâtel, Cognitive Science Centre, Neuchâtel, Switzerland; University of Medicine & Dentistry of NJ - New Jersey Medical School, UNITED STATES

## Abstract

A basic premise in behavioural ecology is the cost-benefit arithmetic, which determines both behavioural decisions and evolutionary processes. Aggressive interactions can be costly on an energetic level, demanding increased energy or causing injuries, and on a psychological level, in the form of increased anxiety and damaged relationships between opponents. Here we used urinary glucocorticoid (uGC) levels to assess the costs of aggression in wild chimpanzees of Budongo Forest, Uganda. We collected 169 urine samples from nine adult male chimpanzees following 14 aggressive interactions (test condition) and 10 resting events (control condition). Subjects showed significantly higher uGC levels after single aggressive interactions compared to control conditions, likely for aggressors as well as victims. Higher ranking males had greater increases of uGC levels after aggression than lower ranking males. In contrast, uGC levels showed no significant change in relation to aggression length or intensity, indicating that psychological factors might have played a larger role than mere energetic expenditure. We concluded that aggressive behaviour is costly for both aggressors and victims and that costs seem poorly explained by energetic demands of the interaction. Our findings are relevant for studies of post-conflict interactions, since we provide evidence that both aggressors and victims experience a stress response to conflict.

## Introduction

A central principle in behavioural ecology is that the function and evolution of behaviour is analysed in terms of economic logic as costs and benefits, which determines an individual’s fitness [1, 2]. Cost-benefit analyses are regularly used to predict how animals should behave to maximize their net fitness gains, including during fights [3, 4]. Despite its importance, it has been surprisingly difficult to quantify the true costs and benefits of a behaviour. One approach is to look at physiological variables, such as hormones secreted in response to stressful events. Although the stress response is generally adaptive, high glucocorticoid (GC) levels are thought to be costly for animals, so measuring their prevalence in wild populations is of importance [5, 6]. GC release, whether it is part of the stress response to energetic stressors [7, 8] or to psychological stressors [9], is costly due to the subsequent release of energy reserves, otherwise needed to support reproduction and growth [10]. Additionally, a range of costly and negative health effects, including impaired immune and cognitive functions, have been linked to chronic release of GCs [11, 12]. Overall this suggests, that GC levels are a reliable proxy for costs, whether the release of GC is caused by energy demands [13–14] or by psychological effects, such as anxiety, uncertainty or relationship damage [15–19], (for review: [6]).

The relationship between high GC levels and aggressive behaviour has been established in a wide range of taxa (fish: *Oncorhynchus mykiss* [7]; reptiles: *Urosaurus ornatus* [20]; birds: *Parus major* [21]; mammals: *Canis lupus* [16], *Cavia porcellus* [22], *Helogale parvula* [16]; primates: *Macaca assamensis* [23], *M. sylvanus* [24], *Pan troglodytes* [14, 17], *Homo sapiens* [8, 25]). In most cases, these studies correlated aggression rates and GC levels over long sampling periods, making statements about causality difficult. Exceptions include several studies that have shown an increase of GC levels in plasma following prolonged aggressive interactions (fights over dominance in fish: [7]; Judo or wrestling matches in humans: [8, 25]). One way to determine causality between aggressive behaviour and excretion of GCs is to study the hormonal effect of single events of aggression under natural conditions.

In many non-human primates there is an inverse linear relationship between dominance rank and aggression received [26], resulting in more incidences of aggression towards lower ranking individuals. Consequently, studies have focused on the relation between GC levels and received aggression [23, 24]. However, other studies in humans [8, 25], rodents [22] and fish [7] have examined plasma cortisol increases in both winners and losers of aggressive interactions. In chimpanzees, however, aggressors do not always win [3], so GC levels might increase in both aggressors and victims (receivers) of aggression.

Giving or receiving of aggression affects the subsequent behaviour of opponents. Prior aggression sometimes attracts further aggression [27] and former opponents seem to avoid each other, if the aggression stays unreconciled [28]. These effects have been attributed to post-conflict anxiety and damaged relationships, both psychological stressors of aggressive interactions. Even though the stress response is likely caused by the resulting uncertainty, any release of GCs will still incur metabolic costs to the individual.

Here, we investigated whether single events of aggression in male chimpanzees (*Pan troglodytes*) caused increased urinary glucocorticoid (uGC) levels in victims and aggressors. We compared uGC levels related to aggression with uGC levels of samples related to pre-aggression periods. We also investigated the effect of duration and intensity of aggression on uGC levels, since fighting duration is correlated with energy use, and contact aggression can more easily lead to injuries than non-contact aggression. Finally we investigated the effects of dominance rank in relation to aggression. Given evidence of reproductive skew in male chimpanzees with dominant males siring more offspring [29, 30], high ranking males arguably have more to lose than lower ranking males.

## Material and Methods

### Study site and data collection

We observed nine adult male chimpanzees (*P. t. schweinfurthii*, **Table 1**) of the Sonso community living in the Budongo Forest (1°35’ - 1°55’ N, 31°08’ - 31°42’ E), Uganda, between February 2008 and July 2010. The Sonso community has been observed continuously since 1990 [31] and was comprised of 15 males (≥15 years: 10; 10–14 years: 5), 35 females (≥14 years: 27; 10–13 years: 8) and 28 juvenile and infants during the study period. Only one adult male was excluded from the study, due to not being sufficiently habituated to tolerate 6 hr focal follows. Budongo forest is a moist, semi-deciduous tropical rain forest with an average altitude of 1100m and a mean yearly rainfall of 1600mm [31].

**Table 1:** Random factors, response and predictor variables tested in the models.

Subject [code]	Year of birth^1^ [yyyy]	Target behaviour [context]	Relative uGC [%]	Duration of behaviour [min]	Rank of subject^2^ [order]	Role of subject [aggressor / victim]	Intensity of aggression [non- / contact]
HW	1993±1y	aggression	104.5	2	7	aggressor	contact
NK	1982±1y	aggression	122.5	1	1	aggressor	contact
SQ	1991±1y	aggression	111.4	1	6	aggressor	contact
ZF	1982±1y	aggression	92.5	1	4	aggressor	non-contact
BB	1987±1y	aggression	146.1	3	2	victim	non-contact
FD	1994±1y	aggression	95.6	2	9	victim	contact
HW	1993±1y	aggression	65.6	3	7	victim	contact
KT	1993±1y	aggression	115.8	3	5	victim	non-contact
MS	1991±1y	aggression	140.6	1	3	victim	contact
NK	1982±1y	aggression	170.1	2	1	victim	contact
NK	1982±1y	aggression	82	2	1	victim	non-contact
SQ	1991±1y	aggression	80.4	3	6	victim	contact
ZF	1982±1y	aggression	128.7	2	4	victim	non-contact
ZF	1982±1y	aggression	107.1	1	4	victim	contact
FD	1994±1y	resting	54.3	30	9		
HW	1993±1y	resting	85.9	115	7		
HW	1993±1y	resting	69.4	60	7		
KT	1993±1y	resting	145.3	125	5		
MS	1991±1y	resting	49.6	45	3		
NK	1982±1y	resting	91.4	50	1		
NK	1982±1y	resting	99.7	30	1		
SQ	1991±1y	resting	87.5	45	6		
TK	1960±5y	resting	82.9	60	8		
ZF	1982±1y	resting	81.3	60	4		

^1^estimated

^2^taken from Table S1 of [38]

With a team of up to six observers, we followed up to three parties of chimpanzees (independently moving subgroups) from approximately 7 a.m. to 5 p.m. through the forest, recording the party behaviour using all occurrence sampling [32] for aggression, grooming and affiliative social interactions. We waited for one of two target behaviours to occur: (1) aggressive interactions, in which one individual (aggressor) attacked another group member (victim) using either contact (hits, bites or tramples) or non-contact aggression (displays, charges or chases); (2) resting, in which one individual had no social interaction for a minimum of one hour and was sitting or lying for at least the first 30 minutes. Aggression events were measured in minutes. After observing a male engaging in one of the target behaviours, we switched to focal animal sampling [32] of that individual for the next 6 hours, collecting every possible urine sample and recording each change in behaviour. Urine was pipetted from plastic bags, when subjects were sitting > 10m high in the tree or from leaf matter when urination occurred on the ground after subjects had moved away. After collecting, urine samples were stored in a thermos flask containing ice and frozen upon arrival in camp, which was within 10 hours after collection. Urine collection did not commence when subjects had engaged in aggression or grooming within the hour prior to the target behaviour, and was aborted when subjects engaged in additional aggression or grooming within two hours after the target behaviour. We collected a total of 169 urine samples (aggression context N = 94, resting context N = 75) from nine adult male chimpanzees following 14 aggression events (9 contact and 5 non-contact) and 10 resting events, with a mean of 7.04 urine samples collected per chimpanzee per target behaviour.

### Hormone analysis

Urinary GC levels were measured at the Lab for field-endocrinology at the Max Planck Institute for Evolutionary Anthropology using high-performance liquid chromatography-tandem mass spectrometry (LC-MS/MS), applying a method that measures 23 endogenous steroids in small quantities of primate urine [33]. Samples with a recovery of the internal standard deviating by less than +/- 50% from the expected value were included in the analysis. In case of large deviation we re-measured the samples. If a large deviation persisted, we re-extracted and re-measured the samples. We excluded samples where the large deviation still persisted. Examination of LC-MS/MS data was carried out with MassLynx (version 4.1; QuanLynx-Software). Only a fraction of plasma cortisol can be found in chimpanzee urine [34], while metabolites of cortisol are found in higher quantities [33]. To quantify the urinary glucocorticoid excretion (uGC), we used the sum of urinary cortisol plus four of its metabolites (tetrahydrocortisol, tetrahydrocortisone, 5β-androstane and 11-oxoetiocholanolon). The sum of uGC comprised on average 9% cortisol, 37% tetrahydrocortisol, 35% tetrahydrocortison, 5% 5β-androstane and 14% 11- oxoetiocholanolon. We corrected the uGC levels with the creatinine levels of each sample to control for differences in water content of urine samples [35]. We excluded samples with a creatinine level of less than 0.05 mg creatinine / ml urine from the analysis.

### Hormonal and behavioural data analysis

**Fig 1:**
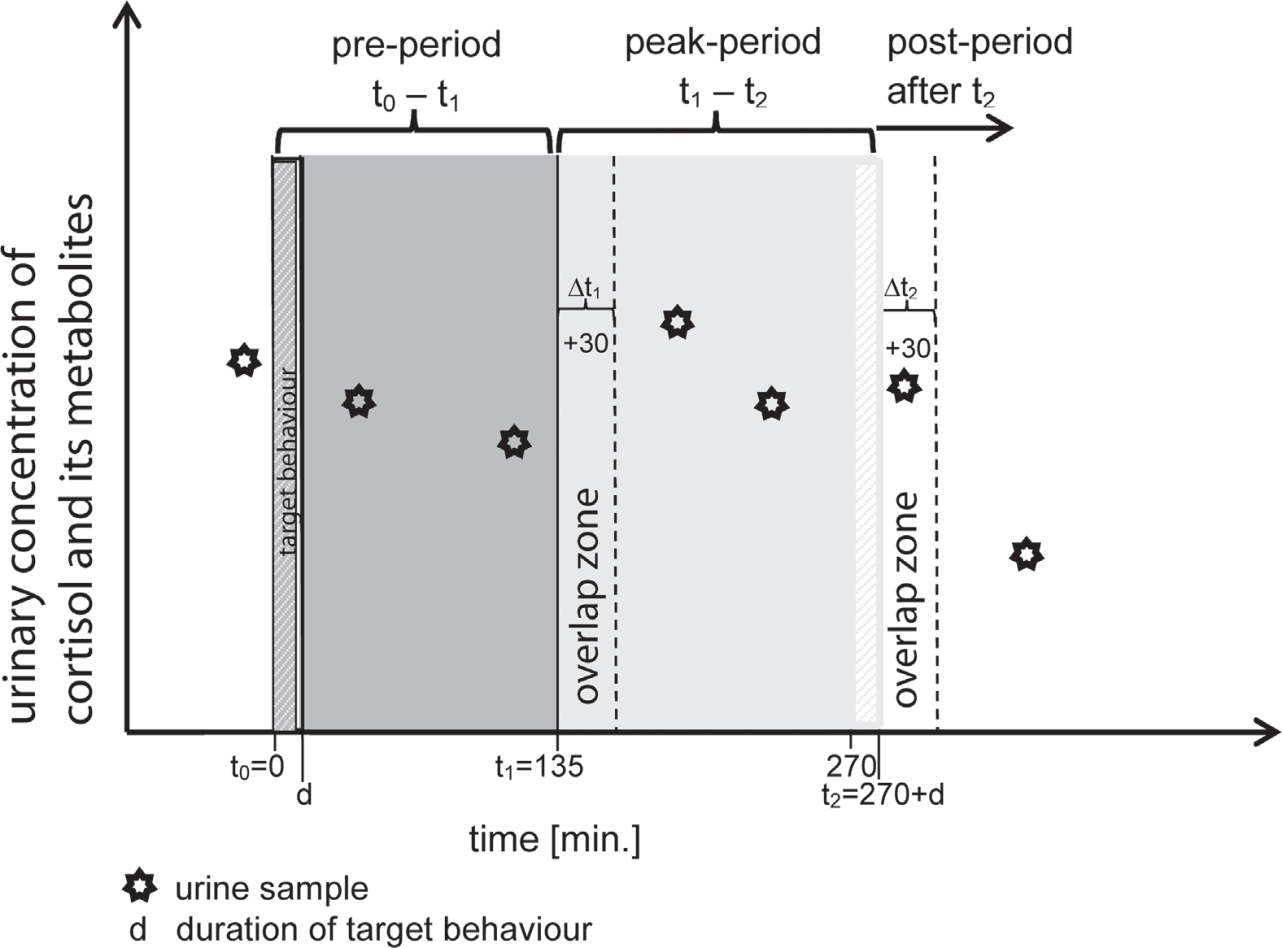
Visual concept of how we classified the different urine samples to the different time periods. The time line is marked in relation to the start of the target behaviour. Point t_1_ refers to 135 min after the onset of the target behavior; point t_2_ refers to 270 min after the end of the target behaviour. The overlap zones correspond to 30 min after t_1_ and t_2_ respectively.

The adult males observed during our study had an average urination interval ± SD = 78 min ± 32. We defined GC clearance in urine of chimpanzees following the results of [34]. Using ^3^H-labeled cortisol, the peak recovery of ^3^H-labeled cortisol metabolites in chimpanzee urine was between ∼2 and 4.8 hours after administration [34]. Although some cortisol was most likely secreted into urine earlier, the highest level of radioactivity recovered was found in the second urine sample at 4.8 h after injection. Based on these finding and due to the variation in the urination interval, we set the window for peak GC clearance in chimpanzee urine to 135–270 min after onset of the target behaviour. Due to diurnal decline in uGC levels [36], absolute uGC levels were not comparable. Thus, we calculated a *relative* uGC level where we divided the uGC level of samples related to the target behaviour (peak-period samples) through the uGC levels of samples collected in the pre-target behaviour times window (pre-period samples). The pre-period urine samples were excreted between 0 min (t_0_) and 135 min (t_1_) after the start of the target behaviour, representing the peak uGC secretion of behaviour preceding the aggression or the resting. The peak-period urine samples were excreted between 135 min after the start (t_1_) and 270 min after the end of the target behaviour (t_2_ = 270+d min, with d = duration of target behaviour; **Fig. 1**), representing the peak uGC secretion of the aggression or resting event. We calculated the relative uGC level for each event, representing the percentage of how high the uGC level was during the peak-period in comparison to the uGC level during the pre-period. Therefore, we divided the mean hormone level of peak-period samples through the hormone level of the pre-period samples: relative uGC level = (mean uGC peak period / mean uGC pre period) x 100 for each subject for each series of urine samples (**Fig. 1**). However, since urine is stored in the bladder and excreted after varying latencies, some urine samples were expected to contain urine from the two adjacent time periods. To address this problem, we assigned these samples to the time period in which the greatest proportion of urine was likely to have been excreted (**Fig. 1**). Specifically, if the sample was collected within 30 min after the pre-period (t_1_) or the peak-period (t_2_), this was considered to be a potential ‘overlap zone’ (**Fig. 1**). ‘Overlap-zone’ samples could be assigned to periods on either side of the period changing point depending on the latency between the sample excreted in the ‘overlap zone’ and the previous urination. If Δt between the sample in the ‘overlap zone’ and t_1_ (or t_2_ respectively) was smaller than between the last sample before t_1_ (or t_2_) and t_1_ (or t_2_), we classified the sample in the ‘overlap zone’ as a sample from the period before t_1_ (or t_2_ respectively). In all other cases the sample was classified as belonging to the time period in which it was excreted (**Fig. 1**).

### Statistical analysis

In order to examine the influence of various predictor variables on changes in urinary GC levels, we ran General Linear Mixed Models (GLMM: [37]) using R version 3.1.1 (R Core Team 2013) and the function lmer of the package lme4 [38], with Maximum Likelihood estimates and a Gaussian structure. Assumptions of normally distributed and homogenous residuals were fulfilled, shown by visual inspection of qq plots and residual plots against fitted values. We checked for model stability by excluding subjects one at a time from the data and state in the results when models were unstable. Variables did not exhibit problems of collinearity [39, 40] (Variance Inflation Factor < 2 in all cases, derived using R-package car [41], applied using a standard linear model excluding the random effect), suggesting that each predictor variable accounted for a portion of the variance.

We ran several GLMMs. In the model including all the data, relative uGC level was the response variable and subject identity was the random factor. We ran additional models which including only the samples relating to aggressive events. This was in order to examine behavioural variation related to aggression (which obviously did not occur for resting events). Due to the small sample size, we were unable to fit all predictor variables into one model. Therefore we looked at the effects of each of the predictor variables on the relative uGC level separately. To establish the significance of the full model we used a likelihood ratio test, comparing the full model with the respective null model comprising only the random effect and the intercept. When models were insignificant, we considered the predictor variables to have no clear effect on the response variable.

### Ethical statement

The ethics committee of the School of Psychology, University of St. Andrews, UK, approved this non-invasive behavioural and hormonal study with the Sonso chimpanzees located around Budongo Conservation Field Station (1°43’ N, 31°32’ E) in the Budongo Forest Reserve, Uganda. In accordance with ethical guidelines we kept 7m distance to the chimpanzees, never interacted with chimpanzee subjects and collected urine with plastic bags, when subjects were sitting > 10m high in the trees, or from leafs after subjects had voluntarily moved. Research was conducted under the permits by the Uganda Wildlife Authority (TDO/33/02) and Uganda National Council for Science and Technology (NS 181). Chimpanzees are an endangered species (IUCN red list) and under the protection of Uganda Wildlife Authority.

## Results

**Fig 2:**
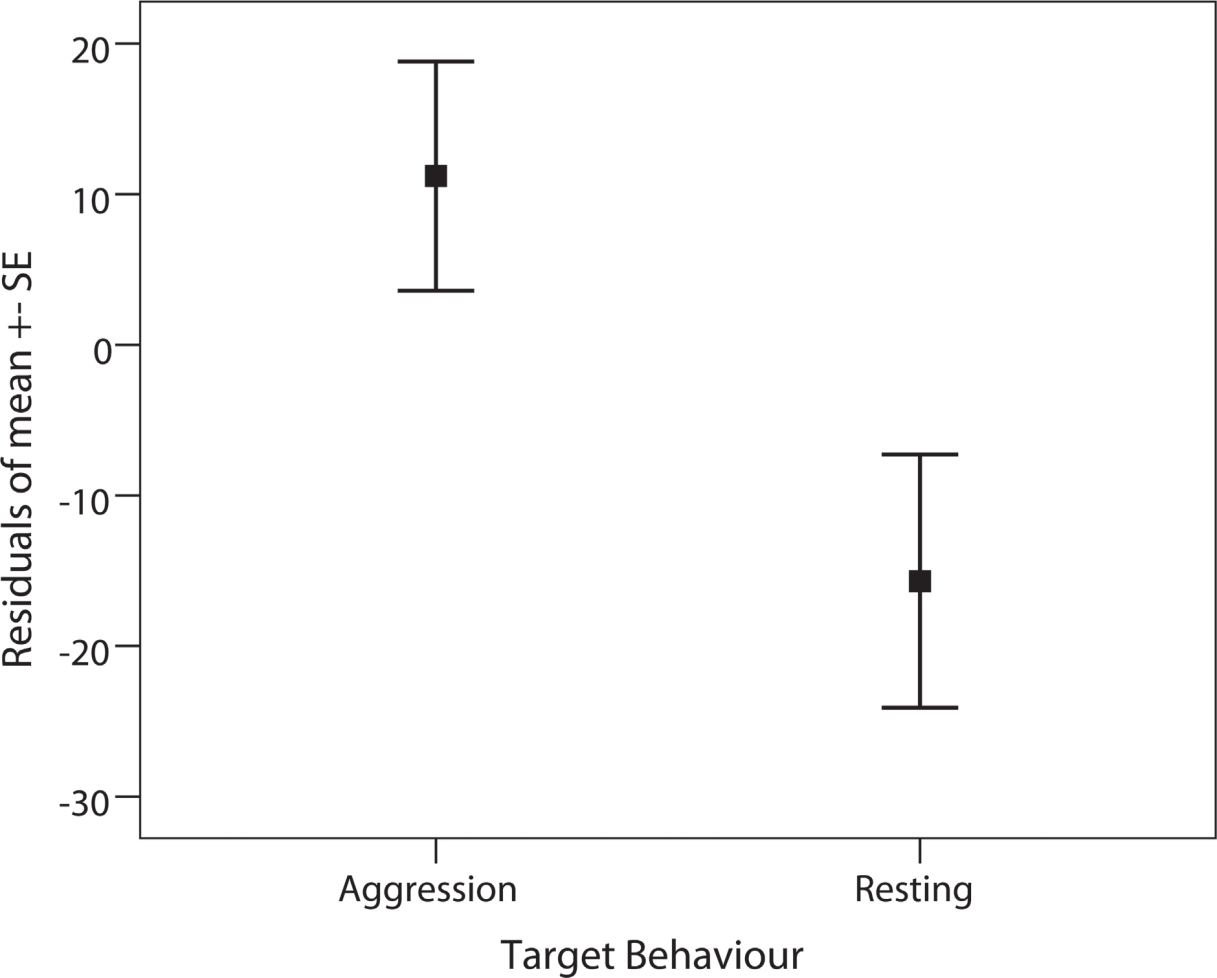
The relationship between the target behaviours (aggression and resting events) and corresponding relative uGC levels, presented as residuals. The relative uGC level is the percentage of the uGC level during the peak-period in comparison to the pre-period.

**Fig 3:**
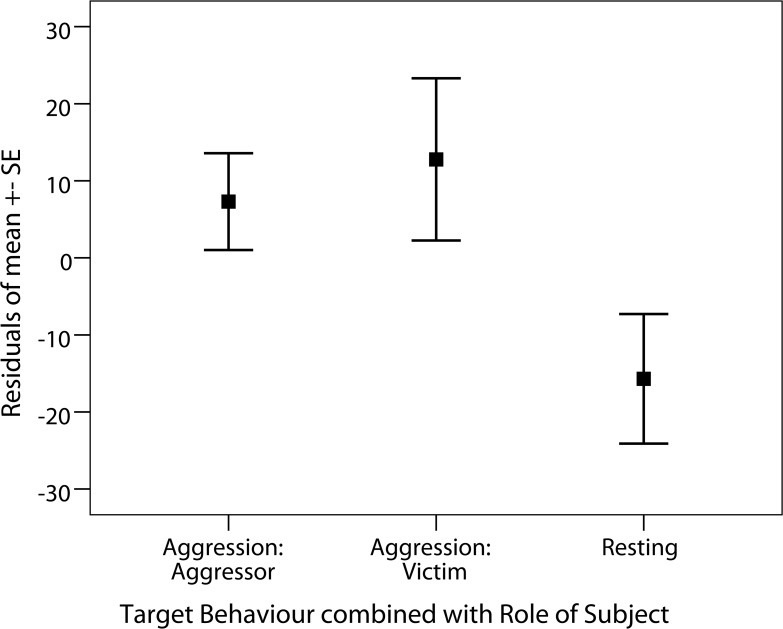
The relationship between the target behaviours combined with the role of subject (aggression: aggressor, aggression: victim, and resting: control) and corresponding relative uGC levels, presented as residuals. The relative uGC level is the percentage of the uGC level during the peak-period in comparison to the pre-period.

Our data set comprised of 14 complete sets of urine samples, where we successfully collected both pre- and peak-samples, following aggression and 10 complete sets following resting (Table 1). Results of the GLMM which included all data showed that the target behaviour (aggression and resting) significantly influenced the relative uGC levels (Table 2A; likelihood ratio test: *χ* ^*2*^ = 5.35, *df* = 1, *p* = 0.021). Males’ relative uGC levels were significantly higher after aggression (mean relative uGC ± SD = 112% ± 28) than after resting for >30 min (mean relative uGC ± SD = 85% ± 27; **Fig. 2**). Combining the target behaviour with the subject’s role during the aggression (aggression: aggressor, aggression: victim, and resting) showed that uGC levels tended to be higher (Table 2B; likelihood ratio test: *χ* ^*2*^ = 5.48, *df* = 2, *p* = 0.065) in both aggressors (mean relative uGC ± SD = 107% ± 12) and victims (mean relative uGC ± SD = 113% ± 33; **Fig. 3**) than after resting. Estimates show that whilst differences in uGC levels were large for both aggressors and victims against resting, differences were small between aggressor and victim conditions (Table 2B).

**Table 2:** The effect of (a) target behaviours and (b) target behaviour combined with subject role on urinary glucocorticoid levels in chimpanzees.

(a)							
Variable	*df*	*χ2*	*p*	Parameter	*β*	*SE*	*t*
**Target behaviour**	**1**	**5.35**	**0.021**	**Aggression (test)**	**26.91**	**10.99**	**2.45**
				**Resting (control)**	**0**		
							
**(b)**							
**Variable**	***df***	***χ2***	***p***	**Parameter**	***β***	***SE***	***T***
**Target behaviour and role of subject**	**2**	**5.48**	**0.065**	**Aggression: aggressor**	**23.00**	**15.66**	**1.47**
				**Aggression: victim**	**28.47**	**11.84**	**2.41**
				**Resting: control**	**0**		
				***Aggression*: *aggressor***	***5.48***	***15.66***	***0.35***
				***Aggression*: *victim***	***0***		

Estimates from variables in italics were taken from a re-run of the model.

**Fig 4:**
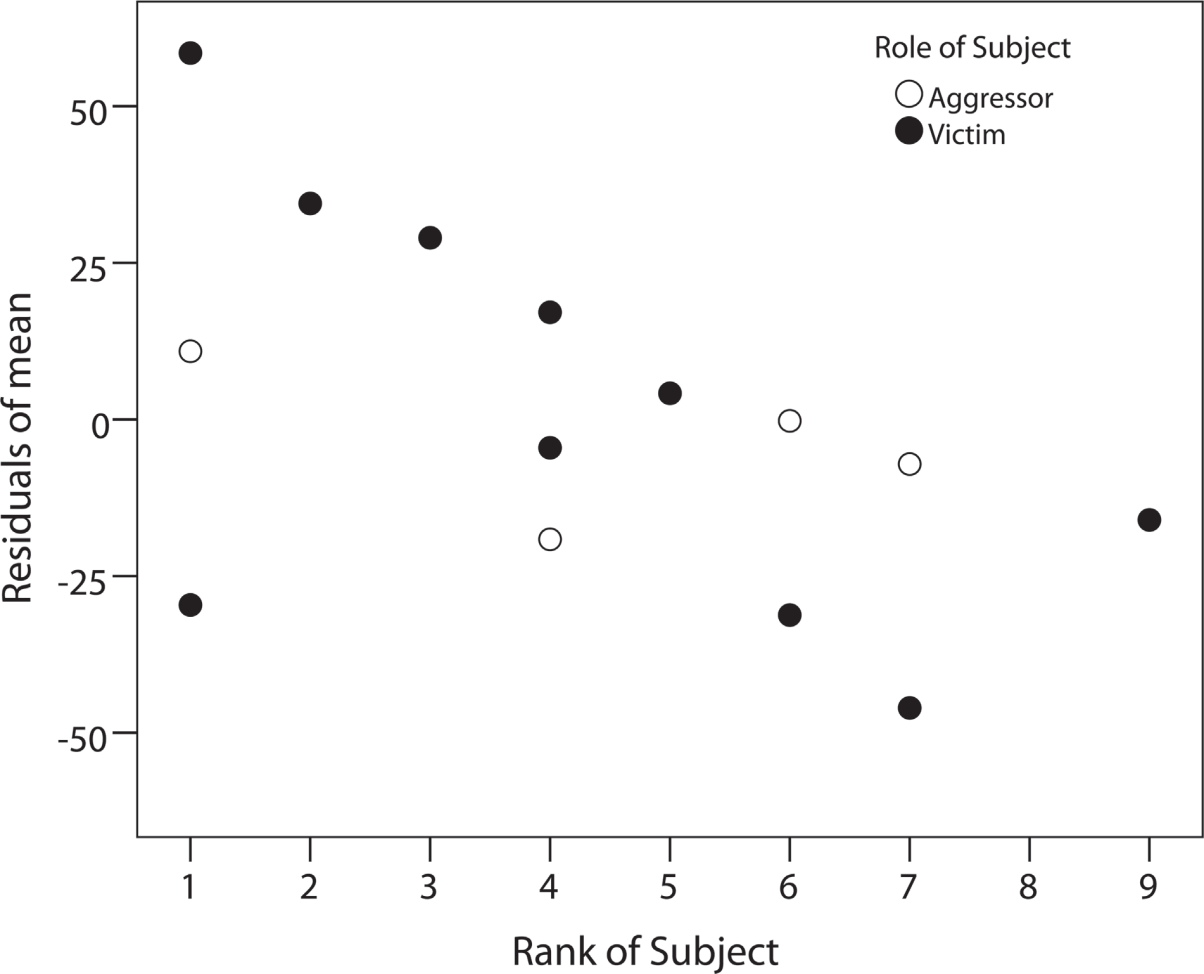
The relationship between subject’s rank and corresponding relative uGC levels, presented as residuals. The relative uGC level is the percentage of the uGC level during the peak-period in comparison to the pre-period. Highest dominance rank = 1.

Further, we investigated in three separate GLMMs whether the subject’s rank (rank 1–9), aggression duration (min.) or aggression intensity (contact or non-contact aggression) affected the relative uGC levels. Only subject’s dominance rank showed a significant effect (**Table 3**; likelihood ratio test: *χ* ^*2*^ = 5.25, *df* = 1, *p* = 0.022) with higher ranking males showing greater elevation of relative uGC levels after aggression (**Fig. 4**). In contrast we did not find effects due to aggression intensity (contact or non-contact) or aggression duration (min.), although it should be noted that negative effects could be a result of small sample sizes (Likelihood ratio tests: aggression duration: *χ* ^*2*^ = 0.45, *df* = 1, *p* = 0.50; aggression intensity: *model unstable*).

**Table 3:** The effect of subject’s rank on urinary glucocorticoid levels in chimpanzees.

**Variable**	***df***	***χ2***	***p***	**Parameter**	***β***	***SE***	***t***
**Subject's rank**	**1**	**5.25**	**0.022**	**rank**	-**5.13**	**2.12**	-**2.42**

## Discussion

Male chimpanzee aggressors and victims had larger increases in uGC levels after single aggressive interactions than after resting. In addition, higher ranking male opponents had greater increases in uGC levels than lower ranking males. Sample sizes were small but our results are comparable to previous studies showing raised plasma cortisol in both winners and losers in humans [25], rodents [42] and fish [7], suggesting that for male chimpanzees aggressive interactions are costly, regardless of the individual’s role in the conflict.

The proximate reasons for the increased uGC levels in our study remain unclear. In fish, plasma cortisol increased after fights in both winners and losers, but winners showed only increased plasma cortisol levels 5 min after the end of the fights, while losers still had increased plasma cortisol levels 24h after the fight [7]. If injury to losers could be ruled out, this study suggests different underlying mechanisms, which may involve energetic costs in winners in addition to a social stress reaction in losers [10]. In another study, chimpanzees showed increased uGC levels prior to hunting events and boundary patrols [43]. The author concluded that elevated uGC levels were in response to the anticipation of the cooperative and/or competitive events [43]. Following this idea, chimpanzees might anticipate the energetic needs of specific behaviours required in the near future. In our study, therefore, it might be possible that aggressors secreted cortisol to release sufficient energy for the attack, while victims primarily released cortisol in response to the psychological stressor of being attacked [26].

However, we suggest this is unlikely to be the only possible explanation, for the following reasons. First, a study in humans has shown that competitive martial arts matches release significantly more plasma cortisol than comparable non-competitive exercise [8], suggesting that uGC rises can be linked to a psychological component induced by competitive contexts. Second, we were unable to detect a significant relationship between the duration or intensity of aggression and the uGC elevation, although longer and more intense fights should have consumed more energy. This suggests that in chimpanzee conflicts, psychological rather than energetic stressors seem to have a greater influence on GC levels. This conclusion, however, needs to be treated with caution due to the small sample size.

With the above in mind, it is very unlikely that the energetic stress hypothesis [13] alone can explain why higher ranking male opponents had a greater increase in uGC levels than lower ranking ones. This is especially true, because our study shows greater increases in uGC levels in reaction to the aggression, rather than high absolute uGC levels. Another possible explanation comes from baboons, where dominant males showed higher fecal GC levels during times of social instability [44]. Social instability, the time of rank changes and male immigration, was also strongly correlated with drastically increased aggression. However, rates of aggression, given or received, did not correlate with fecal GC levels [44], again suggesting that GC level increases were not well explained by energetic costs. Rather, dominant male baboons had more to lose than subordinate ones when considering Chacma baboons’ strong reproductive skew. Reproductive skew is less pronounced in chimpanzees, but is nonetheless present [29, 30]. Thus, high ranking male chimpanzees likely have more to lose from defeat than low ranking males. As a result the GC reactivity due to psychological stressors might be stronger in high ranking than in low ranking males.

Social stress is often caused by a lack of control and predictability, such as the exclusion from food or other resources, or the threat of recurring aggression [6, 11]. This is particularly pertinent in chimpanzees, with their fission-fusion social structure that leads to constantly changing networks of social support, so that even dominant individuals can lose aggressive interactions [3, 45, 46]. Powerful aggressors cannot be certain of winning a conflict, and thus lack a degree of control and predictability. This may be a general pattern in animal groups with more egalitarian social hierarchies and may provide an explanation for increased uGC levels after aggression in aggressors as well as victims.

Our results further our understanding of conflict management in animals. Several studies on primates have shown an increase of self-directed behaviours in aggressors and victims [47–49], (but: [50]), suggesting that both suffered from anxiety after aggression [51, 52]. Traditionally, however, it was assumed that victims, not aggressors, suffer from post-conflict stress. For example, in a study of reconciliation in human boys [53], victims showed higher salivary cortisol levels following unreconciled aggression compared to reconciled aggression or following control conditions [53]. In chimpanzees, ‘consolation’ of victims has been shown to reduce self-directed behaviour [54]. However, these studies have not looked at hormonal responses of the aggressors, as it has not been recognized that aggressors might also suffer a stress reaction. The current study suggests that it is time to reconsider this assumption. Indeed, both victims and aggressors are known to engage in stress-reducing post-conflict coping strategies, such as reconciliation, ‘consolation’ or third-party mediated reconciliation [39, 48, 49, 55].
